# Case report of renal cell carcinoma in automobile manufacturing factory worker due to trichloroethylene exposure in Korea

**DOI:** 10.1186/s40557-015-0068-x

**Published:** 2015-08-03

**Authors:** June-Hee Lee, Inah Kim, Hongdeok Seok, Inhyo Park, Jungho Hwang, Jae-Oh Park, Jong-Uk Won, Jaehoon Roh

**Affiliations:** Graduate School of Public Health, Yonsei University, 50 Seongsanno (134 Sinchon-dong), Seodaemun-gu, Seoul, South Korea; The Institute for Occupational Health, Yonsei University College of Medicine, Seoul, South Korea; Department of Preventive Medicine and Public Health, Yonsei University College of Medicine, Seoul, South Korea; Occupational Safety and Health Research Institute, Korea Occupational Safety and Health Agency, Ulsan, South Korea; Department of Occupational and Environmental Medicine, Hanyang University College of Medicine, Seoul, South Korea

**Keywords:** Trichloroethylene, Occupational disease, Carcinoma, Renal cell, Automobiles, Korea

## Abstract

**Background:**

The aim of this paper was report first case of renal cell carcinoma developed in a worker who worked in an automobile manufacture line which handles trichloroethylene in Korea.

**Case presentation:**

To clarify the relationship between the onset of renal cell carcinoma in 52-years old male worker and the exposure to trichloroethylene, document studies and work environment measurement were done. Past work environment exposure data were reviewed and medical history and surgery records of the worker were also reviewed. The patient had no personal risk factor related to renal cell carcinoma except for his smoking habit of quarter a pack per day for twenty years, and since trichloroethylene was not part of measurement criteria, past work environment risk assessment data could not verify the exposure. The exposure level is deduced by analyzing material exposure level of work environments which has similar processes in data from revised research of chemical exposure standard and work environment validity assessment. Evaluation Committee of Epidemiologic Survey decided that there are relevant relationship between the exposure and the disease, though we do not have exact data during that period, most experts agree that in every factories they used trichloroethylene without any direction.

**Conclusions:**

From the relevant medical history and the results of the usage of trichloroethylene in the relevant industries, and initial discovery of renal cell carcinoma at health inspection sonogram in 2001, it can be concluded that suggests significant causal relationship between the exposure to trichloroethylene and renal cell carcinoma onset, thus reporting it to be the first domestic case declared to be occupational disease.

## Background

Trichloroethylene is colorless and transparent liquid with luscious scent. Since its usage in dry-cleaning in the 1930s, trichloroethylene was widely put to use in the 1900s. It is an organic solvent which has been widely used for detergent for parts in work environments, drying metal plates, cleaning and drying in fabric industries, common solvent, diluents of lacquer, and removal of synthetic leather fat [1].

For its relatively higher boiling point and excellent cleansing capacity, trichloroethylene was utilized throughout the entire industry. Trichloroethylene is catalyzed by cytochrome p450 enzyme, which is metabolized by bichloride acetic acid, trichloride acetic acid, which is then discharged through kidney, and incurs genetic mutation of kidney cells [2]. Especially, International Agency for Research on Cancer (IARC) considers smoking, X-ray, Gamma ray, trichloroethylene to have sufficient evidences related to renal cell carcinoma [3, 4]. Originally considered to have limited evidence to renal cell carcinoma, trichloroethylene was re-evaluated to have sufficient evidence for renal cell carcinoma in 2012. Other materials considered to have limited evidence are arsenic, organoarsenic, cadmium, and printing processes [5].

In Korea, according to the data from National Cancer Control Institute in 2010, the occurrence trend of renal cell carcinoma increased from crude rate of 3 out of 100,000 in 1999 to 3.6 in 2002, 4.9 in 2005, 5.4 in 2006. In 2010, it reached 7.2 which is more than double the rate in 1999. Since the implementation of cancer registry, it has shown steady increase. Renal cell carcinoma showed the highest increase, ruling out the increase of thyroid cancer and prostate cancer. The crude rate of renal cell carcinoma among men in 2010 was 10.1 out of 100,000, which was the ninth most occurred cancer [6, 7].

However, there have been not any reports regarding exposure to trichloroethylene and hepatotoxicity, reports regarding renal cell carcinoma in Korea [8]. As we have experienced the first case of renal cell carcinoma caused by trichloroethylene which has been approved to be an occupational disease, we will brief with document reviews.

## Case presentation

### Patient information

Fifty-two- year-old-male

### Chief complaint

Renal cell carcinoma on the left kidney discovered at medical check up.

### Present illness

**Fig. 1:**
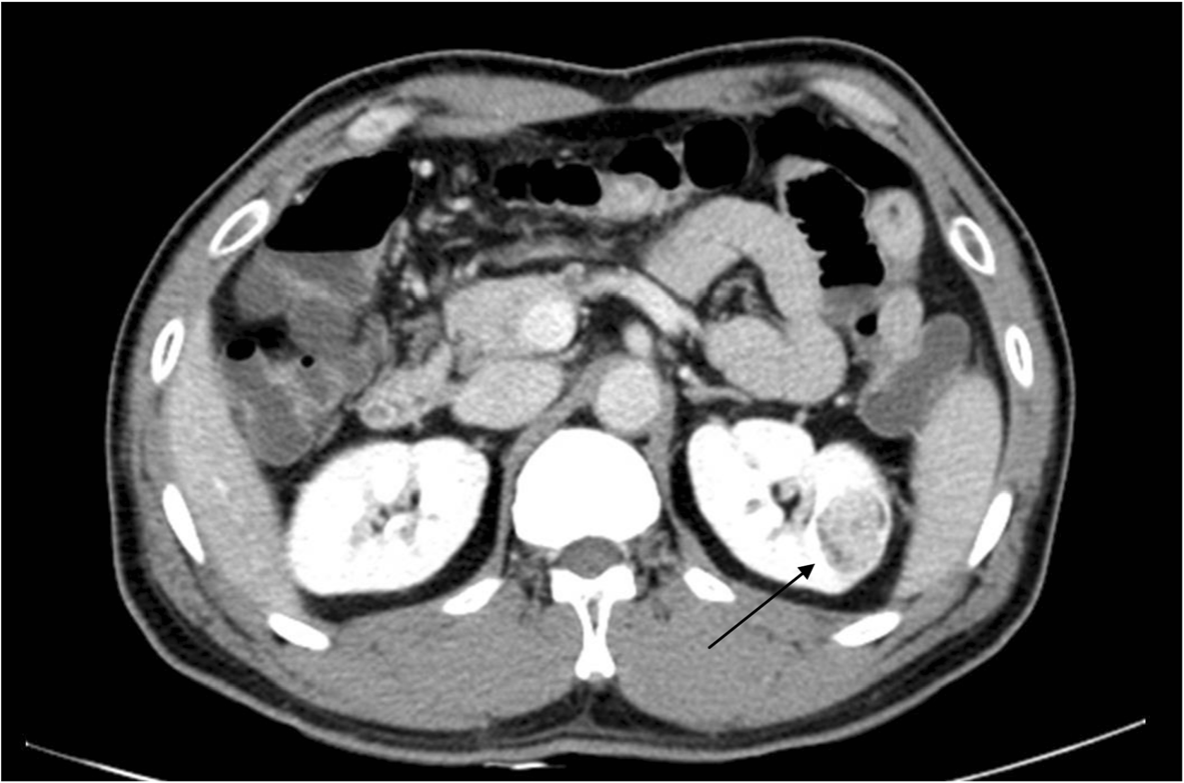
CT scan of renal cell carcinoma (Arrow)

The patient has discovered a cystoma on the left kidney during regular medical check-up in 2003. The size of the cystoma has grown from the original 1.7 cm to 2.7 cm in 2011, therefore, nephrectomy on the left kidney was implemented after CT scan (Fig. 1). Afterwards, through biopsy, the definite diagnosis was renal cell carcinoma. After nephrectomy in 2011, having considered his occupational history to have connection with the renal cell carcinoma, the patient claimed to approve his cancer as occupational disease according to Industrial Accident Compensation Insurance Act.

### Past history

The patient has been diagnosed with type 2 diabetes in 2005, and has received drug treatment until now, and has no outstanding past medical history. The patient has smoked a quarter of a pack of cigarette per day since the age of 26 for about 2 decades and has stopped smoking since 2006. The patient drank three times a month, and each time he drank alcoholic beverage that contains 30 g of alcohol. The patient exercises half an hour a day and has a regular lifestyle. There has been no family history on cancer or other illnesses.

### Occupational history

Employed in 1988, the patient was assigned to do abrasive polishing of the crankshaft forging surface until September 1998, and was assigned to do painting from 1998 to present. The abrasive polishing was manual process for several decades and it was fully automated since 1998. When, it was manually done in the past and there was high temperature steam in the process of surface polishing. The workers were not used proper protective equipments at the time. Moreover, the process of scrubbing the product with cleanser after abrasive polishing was also done manually without personal protective equipments. Afterwards, the patient was assigned to painting process since 1998, and was rotated in various processes including pretreatment, intermediate, intermediate inspection, finish coat, final painting inspection, 4 to 6 months for each process. During the process, the patient did spray painting, cleansing the contaminated part using solvent or thinner, sanding, and touch up. The number of vehicles processed a day is approximately 20, and touch up process on 5 to 6 parts on average per car was administered.

### Exposure assessment

Examining the chemical materials currently under use, mainly trichloroethylene which was re-evaluated to Group1 since 2012 by IARC to be a risk factor to renal cell carcinoma and cadmium which was classified as Group1. As for 4 part painting (pre-coat, intermediate coat, finish coat and fixed painting part), gear parts and regional sample were gathered. Though we checked the results of work environment monitoring on past trichloroethylene exposure level of 4 part of painting process and crankshaft, auto transmission manufacturing (ATM) process, we couldn’t verify the results since proper measurement was unavailable because, trichloroethylene was not target chemical for work environment measurement. However, as for the 3rd factories, the task of control device manufacturing in vehicle factories, we could verify the measurement results on trichloroethylene and carbon tetrachloride in 1994. After measuring the occupational environment assessment levels on past 3rd factories, trichloroethylene exposure was verified and that process is similar for crankshaft, ATM process. After analyzing samples gathered from work environment assessment, trichloroethylene was not found, whereas cadmium levels were 0.0003 mg/m^2^, 0.0002mg/m^3^ in painting intermediate level, 0.0001mg/m^3^ in finish coat level, and not detected in other levels. Other chemicals such as lead, toluene, styrene were extremely minimal or not detected.

### The possibility of exposure to trichloroethylene of workers and exposure level

TCE is one of well known carcinogen through interruption of glutathione pathway and the oxidation process. And TCE also known to be a harmful for the metabolism of glutathione by low dose (under 0.1 mmol/ml in serum level) exposure [9]. To assess the possibility and the level of exposure to a worker, documents and data related to the level of exposure have been reviewed. In Korea, Cho conducted a study in 2007 based on the database on 2004 occupational environment survey established by Occupational Safety and Health Research Institute (OSHRI). The study surveyed 392 work environments already using trichloroethylene and 38 work environments newly using trichloroethylene, of which it selected 103 work environments based on the type of occupation, the number of workers and region. The industry which used the most trichloroethylene a month was automobile part manufacturers of motor vehicle and engine assembly industry which used 23,920 L, followed by computer and electronics manufacture industry (19,596 L), assembly metal product manufacture industry (11,990 L). The industry most exposed to trichloroethylene was automobile part manufacture industry which had the maximum exposure concentration of 49.87 ppm close to the exposure standard (50 ppm). The industry most exposed to trichloroethylene was automobile part manufacture industry which had the maximum exposure concentration of 49.87 ppm close to the exposure standard (50 ppm). In case the work was done manually or semiautomatic, 87 % of the workers were exposed to trichloroethylene, and in case the work was done automatically, only 13 % to the trichloroethylene (Table 1) [10]. And also study on the exposure level and meta-analysis of trichloroethylene in Korea. It was concluded that can be seen working past cleaning and degreasing and have been exposed to high concentrations and low concentrations with long duration of trichloroethylene. Especially, some studies, the concentration of trichloroethylene was reported 100 ppm or more [11].

**Table 1:** Exposure status according to trichloroethylene use in 2006 in Korea

Type of industry	No. of factories	No. of workers	Amount used (L/month)	Concentration range (ppm)
Total	103	390	87,320	-
Chemical	3	19	4116	0.97 ~ 13.26
Plastics & rubber products	1	35	6830	ND ~ 42.63
Primary metal	6	18	6787	2.91 ~ 37.35
Fabricated metal products	24	59	11,990	ND ~ 30.80
Machinery	18	59	3950	ND ~ 48.48
Computer & electronic products	9	62	19,596	0.08 ~ 41.55
Electrical equipment, appliance	10	56	4771	ND ~ 21.29
Transportation equipment	18	30	23,920	ND ~ 49.87
Others	14	52	5360	ND ~ 39.51

(Source: Cho et al. [10], Kim et al. [11])

*ND* Non-detectable, concentration level was lower than the detection limit

Exposure levels in painting process and ATM process of the workers who worked for measuring the results were less than the detection limit trichloroethylene, which is associated with renal cell carcinoma. To make a conclusion about whether to use trichloroethylene, the results of measurement for the environmental assessment of the past were insufficient. However, industrial hygiene specialists in Board of Committee on Occupational Disease Survey are estimated to be generally trichloroethylene previously worked at the time of the ATM process of the job to be used as a cleaning solution and decided exposure levels similar to the process for review of the articles was determined that when workers are exposed to significant levels of trichloroethylene.

### The results of epidemiologic studies

The most important result was a meta-analysis using the 24 series of cohort studies published and case control studies in 2011, the overall relative risk of those exposed to trichloroethylene and those not exposed was 1.27 (95 %CI = 1.13-1.43). The possibility of confounding by smoking is considered low since the odd ratio on lung cancer did not increase [12]. According to recent review about the epidemiologic evidence on the causal relationship between trichloroethylene and renal cell carcinoma, the result of cohort studies were inconsistent, but the results of case–control studies suggested the association [11].

Brüning et al., conducted studies setting 134 patients who received nephrectomy from 1992 to 2000 and 401 patients who received treatments not related kidney in the vicinity as control group. When classifying all the workers associated with cutting and cleansing the metal, which was similar job, as exposed group without environmental inspection on the work environment or definite knowledge of exposure history, the odds ratio was 5.57 (95 %CI = 2.33-13.32). This study also suggested the dose–response relationship between exposure duration and risk for renal cell carcinoma, which, the odds ratio was 3.78 (95 %CI = 1.54-9.28) for less than 10 year exposure duration, 1.80 (95 %CI = 0.67-4.79) for 10 to 20 years, 2.69 (95 %CI = 0.84-8.66) for more than 20 years [13].

In 2006 France, Charbotel et al., utilized trichloroethylene exposure concentration level (ppm) during employment and the length of employment (year) to calculate cumulative exposure level (ppm・year), which is divided into three groups; 1-155 ppm・year as low exposure group (low), 155-355 ppm・year as medium exposure group (medium), more than 335 ppm・year as high exposure group, and in case of having history of being exposed over 15 min to 200 ppm of trichloroethylene concentration level is said to have been peak exposure. The odds ratio according to the level of exposure was 3.34 (95 %CI = 1.27-8.74) in the high exposure group and therefore statistically significant, 1.03 (95 %CI = 0.29-3.70) in medium exposure group, 0.85 (95 %CI = 0.10-7.41) in low exposure group, thus having a dose–response relationship. Especially, the odds ratio of the high exposure group with history of peak exposure was 3.80 (95 %CI = 1.27-11.40) [14].

The recent case–control group studies on trichloroethylene exposure and renal cell carcinoma published by Moore in 2010 also suggested dose–response relationship according to the entire exposure duration, with 13.5 year as cut-off point, in case of exposure under 13.5 years showed 1.89 (95 %CI = 0.84-4.28), in case of exposure over 13.5 years showed 2.25 (95 %CI = 0.95-5.29). The odds ratio in case of exposure under 1080 h was 1.07 (95 %CI = 0.55-2.09) under general classification and 1.22 (95 %CI = 0.48-3.12) under high validity classification. The odds ratio in case of exposure over 1080 h was 2.22 (95 %CI = 1.24-3.99), 2.86(95 %CI = 1.31-6.23) each. After evaluating with cumulative exposure level, in case of below 1.58 ppm・year of cumulative exposure level had the odds ratio of 1.19 (95 %CI = 0.61-2.35) under general classification and 1.77 (95 %CI = 0.64-4.80) under strict classification, and in case of over 1.58 ppm・year had 2.02 (95 %CI = 1.14-3.59) and 2.23 (95 %CI = 1.07-4.64) each [15].

Brusch et al., that one of outstanding points in nephrotoxicity onset of trichloroethylene is that it shows toxicity even with minimal exposure and dose–response relationship is somewhat relatively unclear, and at the onset of tumor, it shows long-term sustainable toxicity by mutagenicity [16].

It can be known that there is a risk factor for renal carcinogenesis both exposure to high concentrations of short duration and long-term exposure low concentration of trichloroethylene as described above. However, industrial hygiene specialists in Board of Committee on Occupational Disease Survey are estimated to be generally trichloroethylene previously worked at the time of the ATM process of the job to be used as a cleaning solution.

## Conclusions

It is believed that the detergent the patient used in the past was trichloroethylene, though it was difficult to confirm whether or not trichloroethylene was used at the time since there are no measurement results of occupational environment. However, through past document studies and domestic estimates, the usage of trichloroethylene used in similar process at the time of the patient’s employment could be confirmed. Also, there are documents considered to be a significant research result on renal cell carcinoma focusing on whether or not the patient was exposed rather than the relationship between exposure level, which would certify the relationship between exposure to trichloroethylene and renal cell carcinoma. The patient worked on metal polishing which frequently used trichloroethylene from 1988 to 1998 and first discovered neoplasm in 2003. This coincides with the period during which the patient is believed to be exposed to trichloroethylene and it is also the latent period (10–30 years) of renal cell carcinoma [17, 18]. The industrial health experts also acknowledged that there are close relationship between the exposure to trichloroethylene and the latent period of renal cell carcinoma. Therefore Board of Committee on Occupational Disease Survey decided that there are relevant relationship between the exposure and the disease, though we do not have exact data during that period, most experts agree that they used trichloroethylene without any direction. The exposure to trichloroethylene is recognized to be an occupational disease as the first case to influence the onset of renal cell carcinoma. Trichloroethylene is a chemical substance that is used extensively from the past to the present. As for the workers who are suspected of being exposed to trichloroethylene considering occupational history, caution is needed in renal checkup and work relevance assessment considering the latent period of solid cancer onset.

## Consent

Written informed consent was obtained from the patient for the publication of this report and all accompanying images.
